# Identification and characterization of a novel hydroxylamine oxidase, DnfA, that catalyzes the oxidation of hydroxylamine to N_2_

**DOI:** 10.1016/j.jbc.2022.102372

**Published:** 2022-08-13

**Authors:** Meng-Ru Wu, Li-Li Miao, Ying Liu, Xin-Xin Qian, Ting-Ting Hou, Guo-Min Ai, Lu Yu, Lan Ma, Xi-Yan Gao, Ya-Ling Qin, Hai-Zhen Zhu, Lei Du, Sheng-Ying Li, Chang-Lin Tian, De-Feng Li, Zhi-Pei Liu, Shuang-Jiang Liu

**Affiliations:** 1 State Key Laboratory of Microbial Resources and Environmental Microbiology Research Center, Institute of Microbiology, Chinese Academy of Sciences, Beijing, China; 2 School of Life Sciences, University of Chinese Academy of Sciences, Beijing, China; 3 High Magnetic Field Laboratory, Chinese Academy of Sciences, Hefei, China; 4 State Key Laboratory of Microbial Technology, Shandong University, Qingdao, China; 5 Division of Life Sciences and Medicine, and Center for BioAnalytical Chemistry, The First Affiliated Hospital of USTC, Hefei National Laboratory of Physical Science at Microscale, University of Science and Technology of China, Hefei, Anhui, China

**Keywords:** *Alcaligenes ammonioxydans* HO-1, Dirammox, heterotrophic and aerobic ammonia oxidation, DnfA, hydroxylamine oxidase, dinitrogen (N_2_) formation, biogeochemical cycling of nitrogen, bacteria, nitrogen metabolism, oxidase, anammox, anaerobic ammonium oxidation, ANME, anaerobic methane oxidation, AOB, ammonia-oxidizing bacteria, Dirammox, direct ammonia oxidation, DNF, dinitrogen-forming, EPR, electron paramagnetic resonance, HAO, hydroxylamine oxidoreductase, HOX, hydroxylamine oxidase, MMOH, methane monooxygenase, NaN_3_, sodium azide, NO, nitrogen oxide, NO_2_^−^, nitrite, PB, phosphate buffer

## Abstract

Nitrogen (N_2_) gas in the atmosphere is partially replenished by microbial denitrification of ammonia. Recent study has shown that *Alcaligenes ammonioxydans* oxidizes ammonia to dinitrogen *via* a process featuring the intermediate hydroxylamine, termed “Dirammox” (direct ammonia oxidation). However, the unique biochemistry of this process remains unknown. Here, we report an enzyme involved in Dirammox that catalyzes the conversion of hydroxylamine to N_2_. We tested previously annotated proteins involved in redox reactions, DnfA, DnfB, and DnfC, to determine their ability to catalyze the oxidation of ammonia or hydroxylamine. Our results showed that none of these proteins bound to ammonia or catalyzed its oxidation; however, we did find DnfA bound to hydroxylamine. Further experiments demonstrated that, in the presence of NADH and FAD, DnfA catalyzed the conversion of ^15^N-labeled hydroxylamine to ^15^N_2_. This conversion did not happen under oxygen (O_2_)-free conditions. Thus, we concluded that DnfA encodes a hydroxylamine oxidase. We demonstrate that DnfA is not homologous to any known hydroxylamine oxidoreductases and contains a diiron center, which was shown to be involved in catalysis *via* electron paramagnetic resonance experiments. Furthermore, enzyme kinetics of DnfA were assayed, revealing a *K*_*m*_ of 92.9 ± 3.0 μM for hydroxylamine and a *k*_cat_ of 0.028 ± 0.001 s^−1^. Finally, we show that DnfA was localized in the cytoplasm and periplasm as well as in tubular membrane invaginations in HO-1 cells. To the best of our knowledge, we conclude that DnfA is the first enzyme discovered that catalyzes oxidation of hydroxylamine to N_2_.

Biological conversion of ammonia and oxidized nitrogen (*i.e.*, nitrate, nitrite, nitrous and nitric oxides, hydroxylamine, and hydrazine) (Fig. 1*A*) plays an important role in natural nitrogen (N_2_) biogeochemical cycling (1). N_2_ gas in the atmosphere, being the largest N_2_ reservoir, was recruited from various N_2_ compounds *via* the currently well-known microbial denitrification, anaerobic ammonium oxidation (anammox) (2, 3, 4, 5), the recently identified Dirammox (direct ammonia oxidation) (6), and nitrogen oxide (NO) disproportion by ANME (anaerobic methane oxidation) (7, 8). Denitrification and anammox play important role in natural N_2_ cycling. Yet, the importance of the Dirammox and the ANME remained to be evaluated. Except for Dirammox, the biochemistry, particularly the enzymes catalyzing dinitrogen-forming (DNF) reaction, had been identified or deduced (Fig. 1*B*).

**Figure 1:**
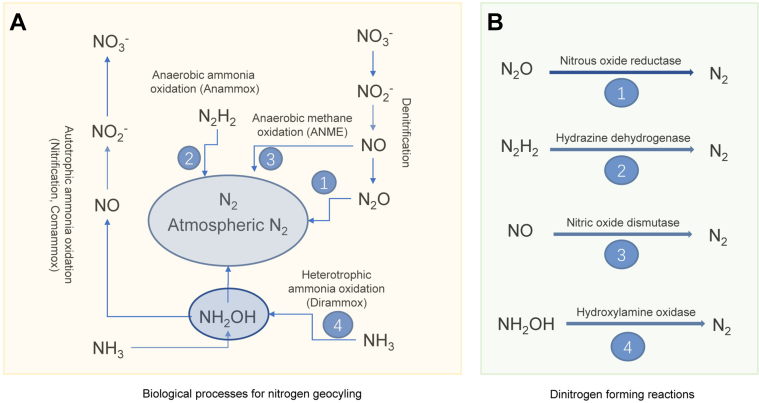
**Microbial processes and biological reactions for nitrogen geocycling in nature.***A*, microbial processes. *B*, biological reactions. References for reactions in *B*: 1—denitrification (2); 2—anammox (4, 5); 3—ANME (7, 8); and 4—this study. ANME, anaerobic methane oxidation.

Hydroxylamine has been identified as a general intermediate from autotrophic or heterotrophic ammonia oxidizers (9). The current understanding indicated that hydroxylamine was converted into NO (NO/N_2_O/NO_2_^−^) during ammonia oxidation, and the reactions were catalyzed by hydroxylamine oxidoreductases (HAOs) (10, 11, 12). HAOs were identified, purified, and characterized from ammonia-oxidizing bacteria (AOB) (13) and anammoxers (14). The canonical HAO from AOB typically oxidizes hydroxylamine to nitrite (NO_2_^−^) (10). Caranto and Lancaster (12) recently demonstrated that NO, not NO_2_^−^, was the obligate product of hydroxylamine oxidation by HAO. The crystal structure of HAO is a homotrimer and each monomer of molecular weight 67.1 kDa and contains seven c-type hemes and a catalytic heme P460 as cofactor in the active site (15, 16). Furthermore, AOB encode a cytochrome P460 (also known as CytL) that detoxifies hydroxylamine by oxidizing it to N_2_O with an unknown biological oxidant (17). Similarly, the hydroxylamine oxidase (HOX) of the anaerobic ammonia oxidizer *Kuenenia stuttgartienses* detoxifies hydroxylamine to NO and thus generates substrate and electrons for respiration (18). Despite the low sequence identity (<30%), this HOX is related and shares structural similarities to the canonical HAOs, of which both HAOs and HOX are homotrimers and have c-type hemes and the P460-type catalytic centers (18). The archaeal HAO is currently unknown, and no genes encoding ammonia-oxidizing bacteria-like homologs were found in archaeal genomes (19). To our knowledge, neither the HAOs nor other enzymes convert hydroxylamine and produce molecular N_2_ as product.

Recently, *Alcaligenes ammonioxydans* HO-1 was found efficiently converting ammonia to N_2_ *via* a newly proposed pathway termed Dirammox (6). A genetic cluster *dnfABC* was found to be essential for this N_2_ production with *A. ammonioxydans*. The genetic cluster *dnfABC* was deduced to encode enzymes that sequentially oxidized ammonia (or its derivatives) to hydroxylamine and to N_2_. In this study, we found that the one of the gene in this genetic cluster *dnfABC*, dnfA, encoded a HOX. Uniquely, this DnfA HOX catalyzed the oxidation of hydroxylamine to N_2_.

## Results

### Bioinformatic analysis of translational products of *dnfABC*, purification, and catalytic activities of DnfA, DnfB, and DnfC from recombinant *Escherichia coli* cells

**Figure 2:**
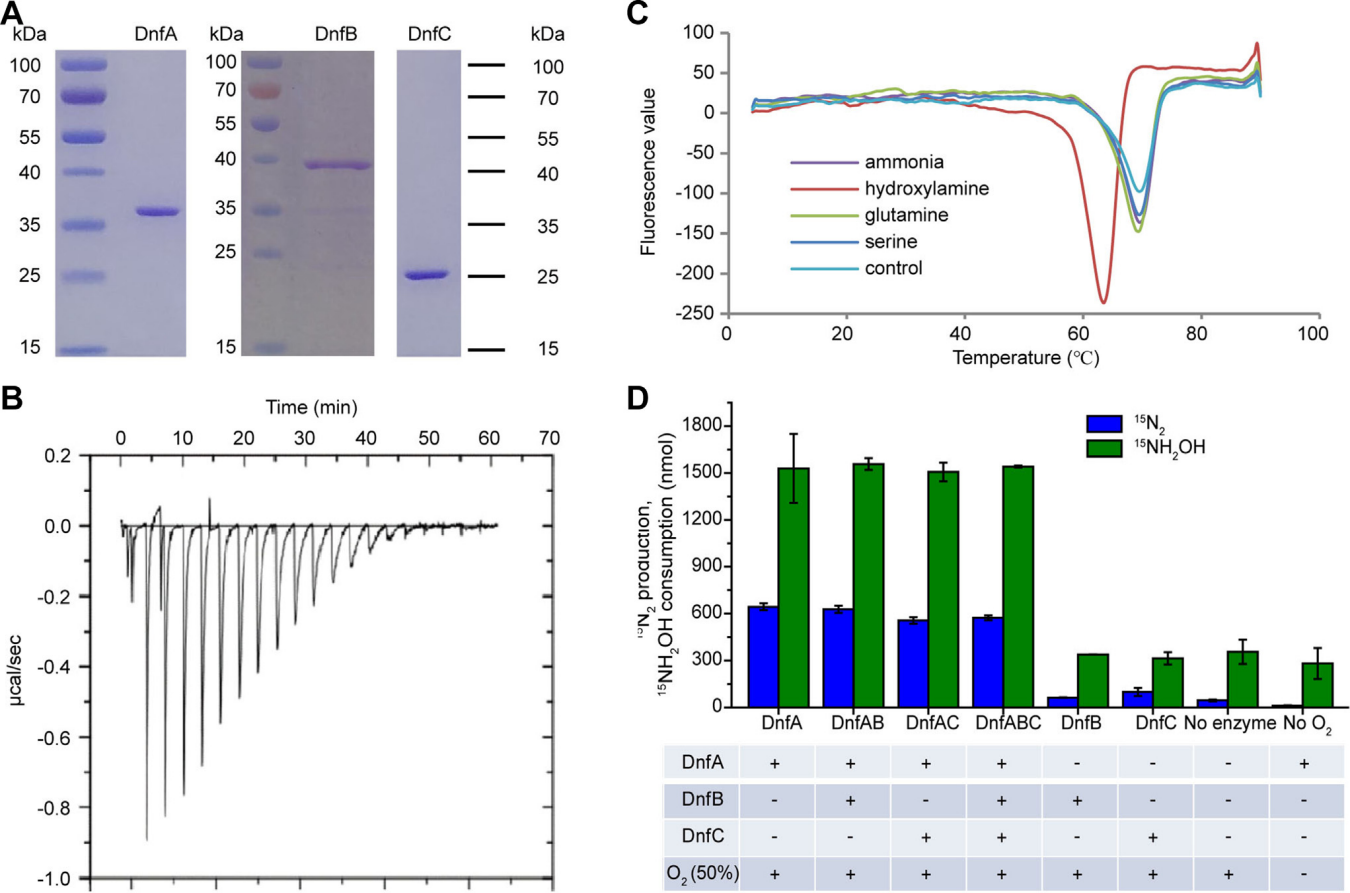
**DnfA bound and converted hydroxylamine to nitrogen.***A*, purification of DnfA, DnfB, and DnfC. *B*, ITC assay of the DnfA–hydroxylamine interaction. *C*, DSF test demonstrated also that DnfA bound hydroxylamine. *D*, enzymatic assay of DnfA activity (331 μM DnfA, 3.2 μM DnfB, and 311 μM DnfC) in the presence of 10 mM ^15^NH_2_OH, 20 μM FAD, 2 mM NADH, 2 mM NADPH, and 50% oxygen (O_2_). Data are averages of three replicates. The assay marked by “no O_2_” is the same as that of DnfA except O_2_. DSF, differential scanning fluorimetry; ITC, isothermal titration calorimetry.

Based on our previous and recent bioinformatic analyses, the translational products of *dnfA*, *dnfB*, and *dnfC* were annotated as diiron oxygenase, NADH-dependent reductase, and glutamine amidotransferase, respectively. *In silico* analyses of DnfA, DnfB, and DnfC did not reveal transmembrane domains or secretion signals and thus most likely these proteins have a cytoplasmic localization (6). DnfA shared up to 28 to 30% sequence identities with the arylamine oxygenase CmlI of *Streptomyces venezuelae* (20), the AurF N-oxygenase from *Streptomyces thioluteus* (21), and CmlI homologs (22) among those documented proteins, suggesting the possibility of DnfA involved in ammonia and/or hydroxylamine oxidation. A diiron motif in DnfA was predicted to consist of 3-His and four carboxylate (Aps/Glu) residues (residues Glu88, Glu123, His126, Glu182, His209, Glu213, and His216) and identical to those in AurF and CmlI (Fig. S1). DnfB was consisted of a FNR-like (ferredoxin reductase) domain that has FAD- and NAD(P)-binding sites and a 2Fe–2S iron–sulfur cluster–binding domain, suggesting its role in shuttling electrons. Since the genetic cluster *dnfABC* conferred *E. coli* cells, the ability to produce hydroxylamine and N_2_ from ammonia (6), first, we individually cloned and expressed *dnfA*, *dnfB*, and *dnfC* in *E. coli*, and the translation products DnfA, DnfB, and DnfC were purified (Fig. 2*A*). The purified DnfA, DnfB, and DnfC were tested for their interactions with ammonia and hydroxylamine. Results showed that none of them interacted with ammonia, but DnfA bound to hydroxylamine (Fig. 2, *B* and *C*). Next, we tested whether DnfA, DnfB, or DnfC was able to catalyze the conversion of ammonia and hydroxylamine. An *in vitro* reconstitution enzymatic activity assay using the chemical electron mediator FAD was performed following the previously reported method for the DnfA homolog AurF (21). We found that neither DnfB nor DnfC catalyzed the conversion of hydroxylamine to N_2_, but DnfA alone did, in the presence of molecular oxygen (O_2_), FAD, and NADH (Fig. 2*D*). The combination of DnfA and DnfB or DnfC oxidized hydroxylamine to N_2_ with this *in vitro* assay systems, but the N_2_ production and hydroxylamine consumption were not significantly different from that of DnfA (Fig. 2*D*). These results indicated that DnfA alone was enough, in the presence of molecular O_2_, FAD, and NADH, for *in vitro* hydroxylamine oxidation, and that DnfB and DnfC were not necessary for the *in vitro* assay. Although a recent study showed that DnfB and DnfC were essential for Dirammox with strain JQ135 (23), how they are involved in the oxidation of ammonia remains unclear.

### DnfA stoichiometrically oxidized hydroxylamine to N_2_ in an *in vitro* reconstitution enzymatic system

Based on the data from *in vitro* reconstitution enzymatic activity assays (Fig. 2*D*), we calculated the stoichiometry of DnfA catalysis. Results showed that the consumption of approximate 2 mol of hydroxylamine produced 1 mol N_2_, suggesting stoichiometric formation of N_2_ from two hydroxylamine N_2_ atoms. Considering that hydroxylamine was completely converted to ^15^N-labeled N_2_ when DnfA was present but not so when DnfA was absent, we concluded that the DnfA-catalyzed reaction, rather than a nonenzymatic reaction, dominated the conversion of hydroxylamine. It was reported that chemical decomposition of hydroxylamine in soils produced N_2_ (24). A trace but detectable amount of N_2_ production was observed in the reaction system without DnfA. A possible explanation might be that O_2_ was activated by FAD/NADH and consequently triggered spontaneous reactions with yet unknown chemistry for N_2_ generation. But the amount of N_2_ production was much lower than the one observed from the DnfA-catalyzed conversion of hydroxylamine (Fig. 2*D*), indicating a major role of DnfA catalysis.

### DnfA single-turnover reaction and the involvement of molecular O_2_ during hydroxylamine oxidation into N_2_

**Figure 3:**
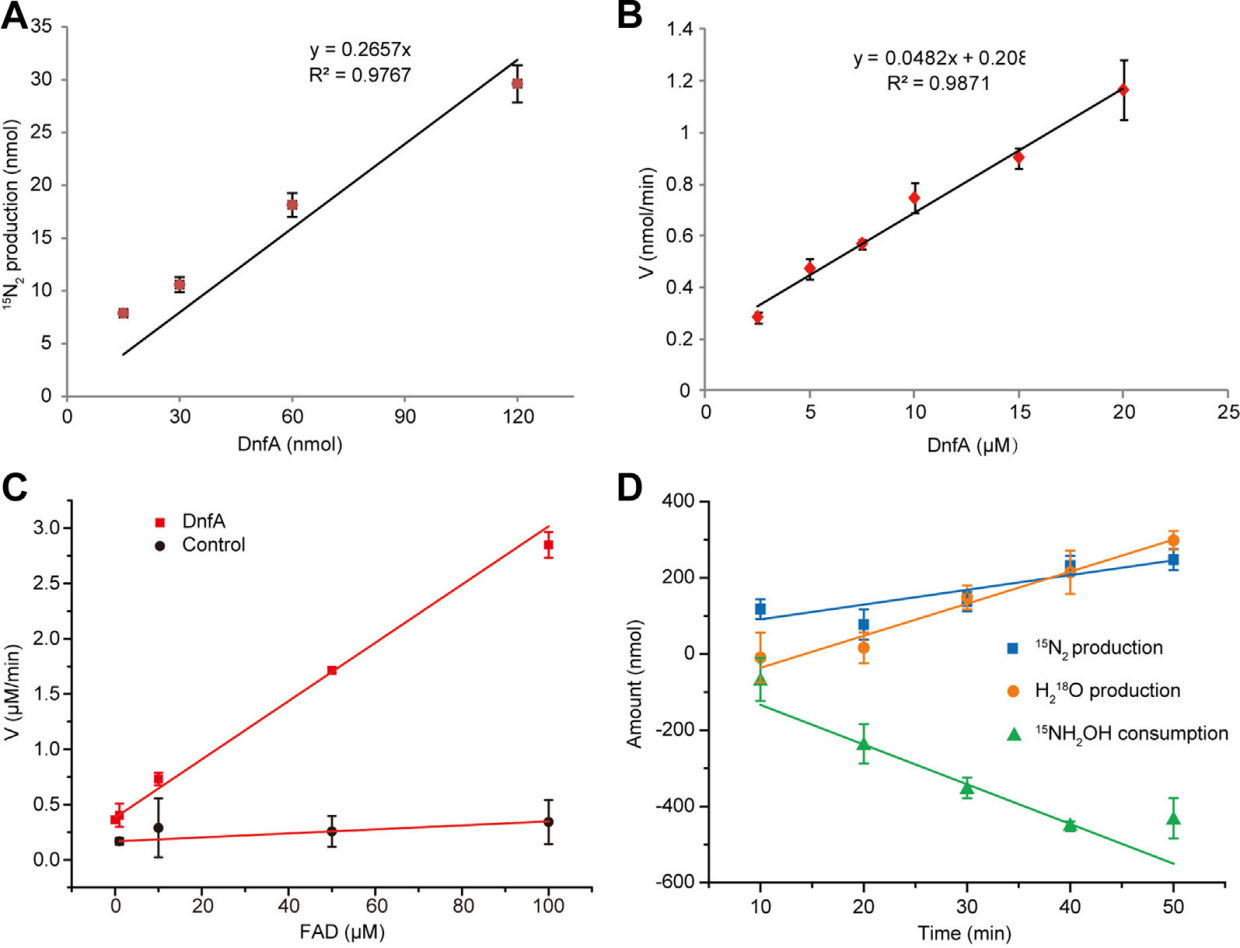
**Cofactors NADH and FAD and molecule oxygen (O**_**2**_**) were involved in DnfA catalysis.***A*, single-turnover reactions of DnfA oxidizing hydroxylamine to nitrogen (N_2_). The assays were performed with initial 1 mM ^15^NH_2_OH, 1 mM NADH, and 100 μM FAD under 20% O_2_ + 80% He. DnfA concentrations were used at 15, 30, 60, and 120 nmol. *B* and *C*, the plots of N_2_ production velocity *versus* DnfA (*B*) and FAD (*C*) concentrations. The assays were performed with initial 1 mM ^15^NH_2_OH, 1 mM NADH under 20 O_2_% + 80 He%. Reaction mixtures without DnfA were used as controls. *D*, molecule O_2_ was involved in DnfA catalysis. Experiments were performed with initial 10 ^18^O_2_% + 90 He% in the head gas space, 10 mM ^15^NH_2_OH, 120 μM FAD, 10 mM NADH, and 330 μM DnfA. The data shown here are the differences of the tests with DnfA and without DnfA, and data are fitted into a linear equation. All tests were carried out in 0.3 ml reactions.

It has been reported that the CmlI and AurF, homologs of DnfA, could catalyze single-turnover reactions in the reduced form (20, 21). We incubated concentrations of as-purified DnfA with ^15^NH_2_OH, and the production of ^15^N_2_ was monitored (Fig. 3*A*). It was found that the production of ^15^N_2_ was quantitatively related to the amounts of DnfA, with a ^15^N_2_:DnfA ratio of 0.26:1 (0.26 N_2_ per diiron cluster). The result indicated that two DnfA molecules catalyzed the oxidization of one molecule of hydroxylamine in the single-turnover reaction. Indeed, we confirmed that native DnfA occurred as dimer with gel-filtration assay. This ratio of 0.26:1 was comparable to that per molecule of activated CmlI converted 0.35 equivalents of substrate (NH_2_-CAM) (20).

We determined reaction velocities at different DnfA concentrations and found that the N_2_ formation velocity was related to enzyme concentrations (Fig. 3*B*). We also determined reaction velocities at different FAD concentrations (0, 1, 10, 50, and 100 μM) with 10 μM DnfA, 1 mM NADH, and 1 mM ^15^NH_2_OH. Results demonstrated that higher FAD concentrations resulted in higher reaction velocities, and 100 μM FAD was appropriate to establish catalytic competence and low background under these enzyme and substrate concentrations (Fig. 3*C*). This mediator FAD concentration was similar with that used in other study (21). We demonstrated that DnfA activity depended on molecular O_2_. By using 10 mM ^15^N-labeled hydroxylamine and 10% of ^18^O_2_ (90% of the gas phase is He), the consumption rate of hydroxylamine was determined to be 0.15 ± 0.02 μM/s per mg protein, and the production rates of ^15^N_2_ and H_2_^18^O were 0.06 ± 0.01 μM/s per mg protein and 0.13 ± 0.01 μM/s per mg protein, respectively (Fig. 3*D*). Another indicted more very solid evidence for O_2_ involvement was that this reaction would not proceed in O_2_-free atmosphere. The molar ratio of hydroxylamine consumption to N_2_ production to H_2_^18^O production was 2.26:1:1.96, a ratio close to the values in Equation 1. We calculated that this reaction is thermodynamically feasible, ΔG^o^’ = −587 kJ mol^−1^.

Based on those results and data, we proposed that DnfA catalyzed the following reaction: (Equation 1).

(1) 215NH2OH+O218+NADH+H+→N215+2H2O+2H2O18+NAD+

### Kinetic constants of DnfA

**Figure 4:**
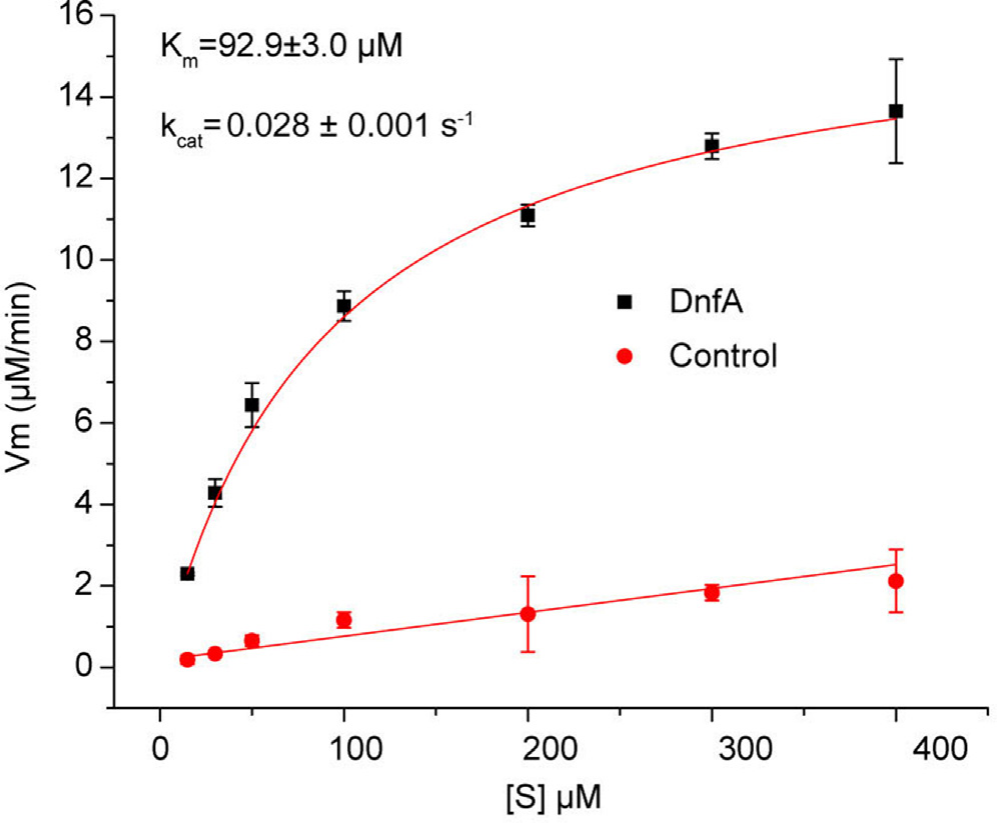
**Determination of kinetic constants of DnfA under atmospheric condition.** The assays were performed with 10 μM DnfA (0 for control assay), 100 μM FAD, 1 mM NADH, and 15 to 400 μM of NH_2_OH at atmospheric condition. The data shown here are averages of three replicates.

Furthermore, the kinetic constants of DnfA-catalyzed reactions at atmospheric condition were determined. Using FAD as the electron mediator, the Michaelis–Menten constant (*K*_*m*_) for hydroxylamine and the catalytic efficiency (*k*_cat_) were determined to be 92.9 ± 3.0 μM and 0.028 ± 0.001 s^−1^, respectively (Fig. 4). Though DnfA converted tlhe hydroxylamine with a relative low catalytic efficiency *in vitro*, the substrate-binding affinity and catalytic efficiency of DnfA are comparable with those reported for the DnfA homolog AurF (*K*_*m*_ = 5.24 ± 0.64 μM and *k*_cat_ = 0.10 ± 0.01 s^−1^). Therefore, hydroxylamine was deduced as the substrate of DnfA under physiological conditions. Moreover, the transcriptional level of DnfA responding to ammonia stimuli was high (6500 ± 400 TPM) and at least one order of magnitude higher than the other genes involved in N-metabolism (6). This high transcriptional level could be a compensation for the low catalytic efficiency.

### DnfA contained iron atom involving in catalysis

**Figure 5:**
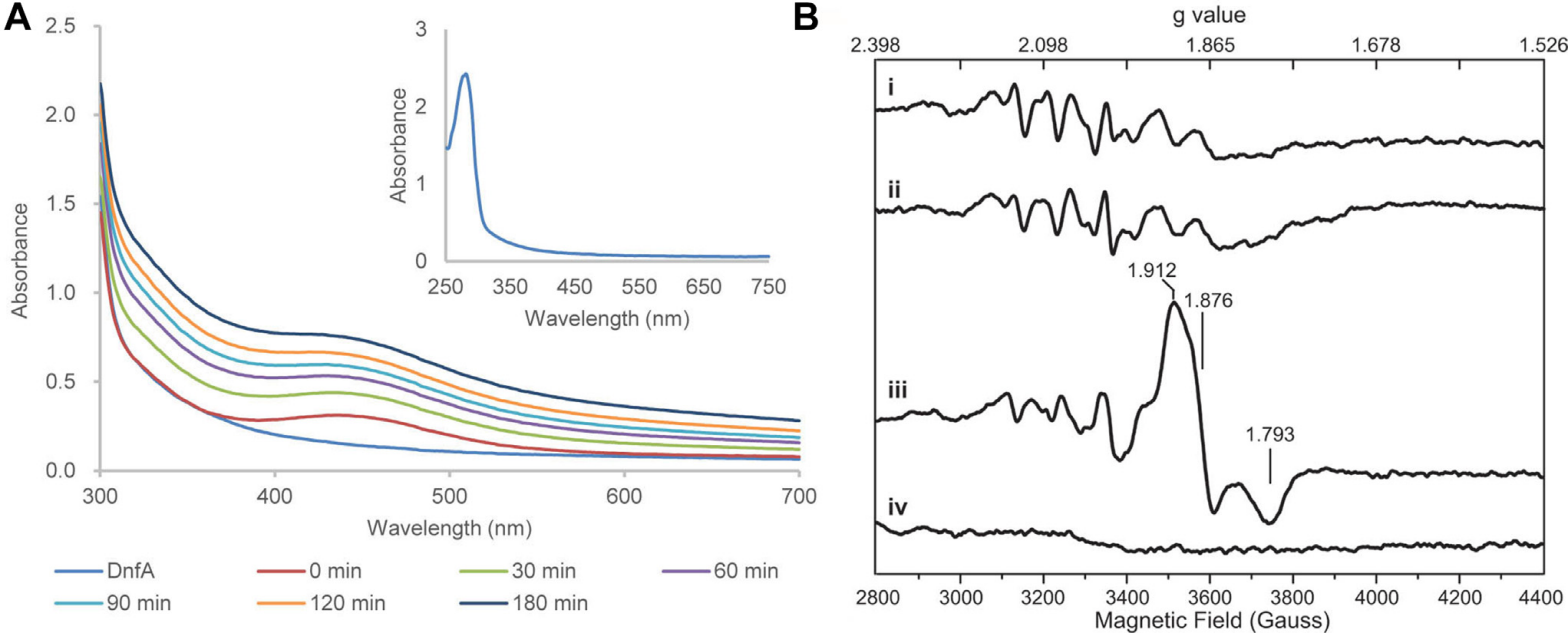
**Spectroscopic characterization of the diiron cluster in DnfA**. *A*, UV–visible absorption spectra of DnfA (*dark blue*) and DnfA treatment with 1 M sodium azide for different time (0, 30, 60, 90, 120, and 180 min). *Inset*, expanded UV–visible absorption spectra of DnfA. *B*, X-band EPR spectrums of as-isolated DnfA (i), DnfA incubated with NADH and FAD (ii), DnfA incubated with NADH, FAD, and NH_2_OH (iii), and dithionite-reduced DnfA (iv) were shown. Numbers indicate the *g* value of each peak. Experimental conditions: temperature, 10 K; microwave power, 2 mW; modulation amplitude, 5 G; modulation frequency, 100 kHz; resonance frequency, 9.397 GHz. In EPR spectrum of DnfA (i), the weak signal observed between magnetic fields of 3000 to 3600 G possibly originates from portion of protein, which took up Mn atoms in place of iron atoms. EPR, electron paramagnetic resonance.

Inductively coupled plasma-MS indicated that iron atoms were associated with DnfA molecules at a ratio of 1.60 ± 0.01:1 and suggested that one molecule of DnfA bound two iron atoms, similar to that AurF and CmlI did (20, 21). These data supported the bioinformatic prediction that DnfA had a diiron motif. The absorption spectrum of DnfA revealed a typical protein feature with absorption at 280 nm (Fig. 5*A*). Usually, a featuring absorption at 300 to 340 nm was observed for binuclear nonheme iron-containing enzymes, which resulted from an oxo-to-Fe(III) charge transfer transition and could be bleached by the addition of reductant reagents (25). The lack of absorption at 300 to 340 nm with DnfA, as well as H_2_O_2_-treated DnfA, suggested that the diiron center might occur as a μ-hydroxo-bridged diferric state instead of a μ-oxo one. In the presence of 1.0 M sodium azide (NaN_3_), a new absorption band of 450 nm was observed for DnfA (Fig. 5*A*). The new absorption band at 450 nm was observed for those enzymes containing diiron clusters in the presence of azide, such as 18:0-ACP delta-9-desaturase, CmlA, and hemerythrin (25, 26, 27). The lack of absorption at 300 to 340 nm and the presence of azide-induced absorption at 450 nm distinguished the diiron center of DnfA with those previously reported diiron center enzymes.

Electron paramagnetic resonance (EPR) experiments were conducted to provide further insights into the catalytic mechanism of this diiron center involved in DnfA catalysis. As shown in Figure 5*B*, the EPR spectrum of as-isolated DnfA (trace i) shows only minor impurities from manganese (IV) species but no signal for the diiron center. This is due to the fact that oxidized cluster [Fe^III^–Fe^III^] has strong antiferromagnetic coupling between the two Fe^III^ atoms and thus is diamagnetic (S_total_ = 0) and EPR silent. Similar EPR spectrum was also observed for AurF in previous studies (28). No change in the EPR spectrum was observed when DnfA was incubated with NADH and FAD (trace ii). However, a new set of rhombic EPR signals (g values at 1.912, 1.876, and 1.793) were observed when DnfA was incubated with NH_2_OH, NADH, and FAD (trace iii), representing a mixed-valent Fe^II^–Fe^III^ state of the iron cluster (28, 29). The EPR spectrum of this state arises from antiferromagnetically coupled Fe^II^ (S = 2) and Fe^III^ (S = 5/2) giving an S_total_ = 1/2 Kramers doublet ground state, which was also observed for mixed-valent monooxygenase hydroxylases (methane monooxygenase [MMOH]), arylamine oxygenase CmlI, and arylamine oxygenase AurF (20, 29, 30, 31). The EPR peaks from both the mixed-valent Fe^II^–Fe^III^ state and the manganese (IV) species disappeared upon the addition of strong reductant dithionite (trace iv), indicating that the protein was fully reduced. Taken together, the observation of the mixed-valent Fe^II^–Fe^III^ state by EPR spectroscopy strongly suggests that the diiron center of DnfA plays crucial roles in catalyzing the transformation of NH_2_OH to N_2_. In addition, the detection of the mixed-valent intermediate state indicates that DnfA might adopt a mechanism involving the diiron center similar to CmlI and AurF, for which the mixed-valent intermediate state was also observed. However, detailed molecular mechanism of DnfA catalysis remains not fully understood and deserves further investigation.

### Subcellular localization of DnfA

As the key enzyme of the novel N_2_ metabolism pathway, the subcellular localization of DnfA triggered our interest. For anammox, the five protein complexes, that is, nitrite oxidoreductase, HAO, hydrazine dehydrogenase, HOX, and hydrazine synthase located at the anammoxosome matrix of *Kuenenia stuttgartiensis* cells, and nitrite oxidoreductase were also related to a tubule-like structure (32). Considering that the quantitatively accumulated hydroxylamine is the substrate of DnfA and it is harmful to other subcellular organic matters, we raised the question if HO-1 forms a membrane-bound structure where the catalytic reaction most likely occurs.

**Figure 6:**
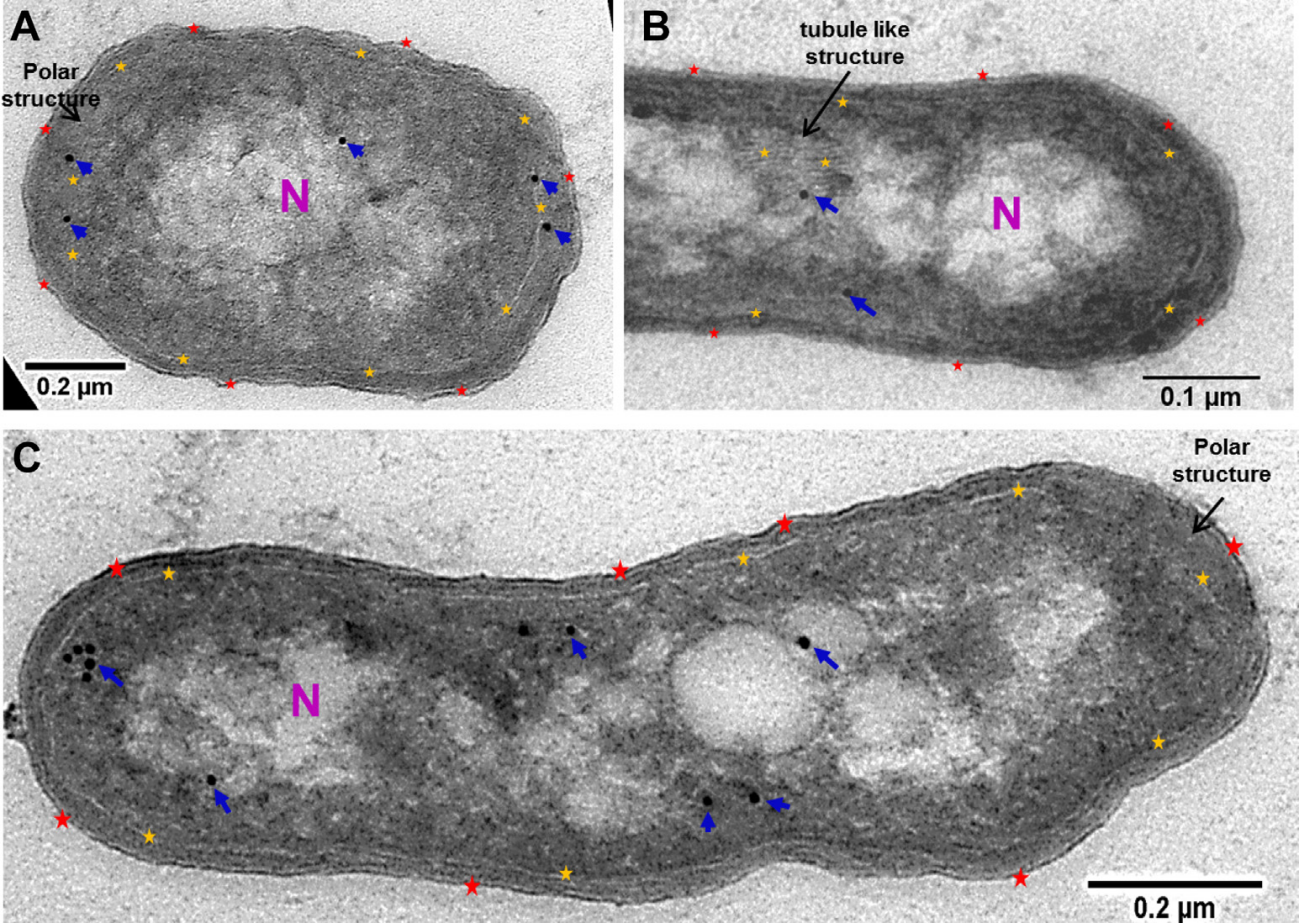
**The immunogold localization of DnfA.***A*, transmission electron micrographs show the ultrastructure of HO-1 (*A*–*C*) and immunogold labeling cryosections incubated with antibodies against DnfA. Gold particles were visible both in the cytoplasm (*A* and *C*), periplasm (*A*), and the tubule-like structure (*B*). The *red and yellow asterisks* indicate the outer membrane and the inner membrane, respectively. Tubule-like structures were indicated by the *black arrow* in *B* on cytoplasm membrane. The *black arrows* in *A* and *C* showed the special morphology at the cell poles. *Blue arrows* point to the 10 nm gold particles. “N” indicated nuclear area.

To explore the subcellular localization of DnfA, the HO-1 cells cultured in HNM medium at 30 °C for 40 h (absorbance at 600 nm = 1.72) were centrifuged and treated immediately to acquire ultrathin sections, labeled with antimouse antibody coupled to 10-nm gold particles, and observed with transmission electron microscope. As shown in Figure 6, the HO-1 cells were uniformly bounded with bilayer (outer and inner) membranes accompanied with the nuclear area in the cytoplasm center, which is the low electron density area in transmission electron microscope photograph. Tubule-like structures were observed in some cells (Fig. 6*B*). We suggested that all cells most probably contained the tubule-like structures, and that the tubule-like structures were visible only if the cell was sectioned through them. The special morphology at the cell poles was observed for approximately 48.6% cells (Fig. 6, *A* and *C*). The structure was proposed to be formed by inner membrane invagination and filled with the same electron density matters as cytoplasm matrix. Labeling studies with goat-derived antimouse antibody (secondary antibody) coupled to 10-nm gold particles showed that DnfA enzyme was located exclusively inside the cytoplasm (Fig. 6, *A* and *C*), periplasm (Fig. 6*A*), and tubule-like structure (Fig. 6*B*). These observations suggested that DnfA did not locate at any special position.

## Discussion

Until now, denitrification and anammox are the major identifiable and biological processes to produce N_2_. In the denitrification process, N_2_O acts as the electron acceptor and is reduced to N_2_ (2). In the anammox process, ammonia and NO form hydrazine (N_2_H_4_), and hydrazine is further converted to N_2_ *via* hydrazine dehydrogenase (5). The oxidation of hydroxylamine to N_2_ was reported abiotically in soil (24). We reported in this communication an enzyme-driven conversion of hydroxylamine to N_2_. N_2_ fixation from N_2_ by N_2_-fixing microbes and anthropogenic processes count for 415 Tg N_2_ fixation per year, and denitrification plus anammox accounted for approximately 350 Tg N_2_ formation per year (1). There are 65 Tg N_2_ difference to make the balance for N_2_ geocycling. A recent survey on riverbed indicated that N_2_ production from denitrification and anammox was lower than the measured N_2_ production, and a cryptic source was contributed to N_2_ production from ammonia oxidation (33). In a recent study, we found a previously unknown process, Dirammox, for aerobic ammonia oxidation, and hydroxylamine was the intermediate (6). In this study, a HOX, DnfA, was purified and characterized from *A. ammonioxydans* HO-1. Based on our studies and the data from Ouyang *et al*. (33), we proposed that Dirammox contributed N_2_ geobiocycling in nature, and hydroxylamine was most possibly the “cryptic source” for N_2_ production from aerobic ammonia oxidation.

DnfA shares less than 30% protein amino acid sequence identity with the diiron N-oxygenases CmlI and AurF and contains a diiron motif of three histidine (His) and four carboxylate (Asp/Glu) residues. This diiron motif of DnfA is identical to those of CmlI and AurF. Both CmlI and AurF catalyze the oxidation of amino group of amines to nitro group (20, 21), but DnfA catalyzes the oxidation of hydroxylamine to N_2_. Therefore, DnfA represents a different category of HOX from CmlI and AurF. This novel reaction catalyzed by DnfA is a previously unknown biochemical reaction mediating the N_2_ formation (Fig. 1*B*). It is particular of note that the enzymes for dinitrogen formation *via* denitrification and anammox are sensitive to O_2_ (2, 34). The DnfA enzyme is rather an O_2_-dependent enzyme, which distinguish this reaction from the previously known N_2_ formation reactions. Although the substrates and products are different for DnfA, CmlI, and AurF, EPR assays showed that the di-iron center of DnfA might switch to a mixed-valent Fe^II^–Fe^III^ state in the reaction similar as the diiron AurF and MMOH did (28, 29). Previous studies revealed the nonheme diiron center of the MMOH is directly involved in the methane oxidation (31). In a recent study, a nonheme diiron N-oxygenase AzoC (a DnfA homolog with sequence identity 30%) was reported to mediate the azoxy bond formation from amine groups (35). AurF and CmlI catalyzed a six-electron oxidation of arylamine to nitroaryl derivatives (20, 21). These studies, besides our previous study, evidenced that DnfA gene was essential of ammonia oxidation and hydroxylamine production (6), imply a possibility of that DnfA was involved in amine or ammonia oxidation as well as the hydroxylamine oxidation and N_2_ formation. Considering that both DnfA and AzoC were involved in the N–N bond formation, we hypothesize that they share a similar mechanism to form the N–N bond. For AzoC, the amine precursor of azoxymycins was oxidized to its nitroso (HNO) analog, mutually converted to the hydroxylamine form by redox coenzyme pairs NAD^+^/NADH *via* a radical transient intermediate, and then interacted with another nitroso group to form the azoxy bond of azoxymycins (35). Thus, we deduce that one molecule of hydroxylamine was oxidized to HNO or a radical transient intermediate at the same oxidation state, further reacted with another hydroxylamine molecule to form a new N–N bond and then lost two water molecules during DnfA catalysis. We are working on the biological ammonia conversion process, as well as the dinitrogen formation mechanism, and advancing the understanding of Dirammox process.

## Experimental procedures

### Purification of DnfA, DnfB, and DnfC

To prepare proteins DnfA, DnfB, and DnfC, *E. coli* BL21(DE3) cells carrying the plasmid pET-21a-DnfA or pET-21a-DnfB and *E. coli* BW25113 carrying the plasmid pBAD-DnfC were grown in LB supplied with 100 μg/ml ampicillin at 37 °C and 200 rpm. When the absorbance at 600 nm reached 0.3∼0.6, *E. coli*/pET-21a-DnfA and *E. coli*/pET-21a-DnfB were induced with 0.5 mM IPTG at 16 °C on a rotary shaker (160 rpm) for 20 h, and *E. coli*/pBAD-dnfC was induced with 0.1% l-arabinose at 16 °C, overnight. The cells were harvested by centrifuging, resuspended in buffer A (100 mM Tris–HCl, 100 mM NaCl, 10 mM imidazole, pH 8.0), and subsequently lysed by ultrasonication at 200 W for about 15 min. After centrifugation at 14,000*g* for 30 min at 4 °C and filtered through 0.45 μM filter, the supernatant was applied to a nickel–nitrilotriacetic acid resin (Qiagen) column, which was previously equilibrated with buffer A. Then the nickel–nitrilotriacetic acid matrix was washed with buffer A by adding imidazole at 10 to 50 mM concentrations to remove impurities. 6xHis-tagged protein was eluted with buffer B (100 mM Tris–HCl, 100 mM NaCl, 250 mM imidazole, pH 8.0). The purified 6xHis-tagged proteins were desalted using centrifugal filter devices (Merck Millipore) and exchanged into buffer C (20 mM Tris–HCl, pH 8.0) with a PD-10 desalting column (GE Healthcare). Protein concentrations were determined by Bradford method using a Quick Start Protein Assay Kit (Bio-Rad). The iron-to-enzyme ratio of purified DnfA was determined by using Inductively coupled plasma-MS quantitative analytical method (28). In addition, gel-filtration assay of native DnfA was performed using Superdex 200 Increase 10/300 GL gel filtration column (300 mm × 10 mm; GE Healthcare). The mobile phase was 20 mM Tris–HCl, 150 mM NaCl buffer (pH 8.0), and UV absorbance at 280 nm was detected.

### UV–visible assays

UV–visible assays were performed according to Makris *et al.* (20). The absorption spectrum was monitored using a multimode plate reader (PerkinElmer, Inc). The spectra range was set from 250 to 700 nm. The protein concentration of DnfA was adjusted to 280 ± 5 μM using 50 mM Hepes buffer (pH 8.0). In order to study the effect of NaN_3_ on the UV spectrum of DnfA, NaN_3_ (4 M in 50 mM Hepes, pH 8.0) was slowly added to DnfA with the final concentration of 1.0 M, and the UV spectrum was detected.

### Differential scanning fluorimetry and isothermal titration calorimetry experiments

Measurements of protein–substrate interactions by differential scanning fluorimetry were carried out in 20 μl reaction mixtures in 20 mM Tris–HCl buffer (pH 8.0) containing 100 μg/ml DnfA, 10 mM small-molecule compound, and 5× SYPRO Orange. Reactions were performed on the real-time fluorescence quantitative PCR instrument CFX96 (Bio-Rad) with the following procedure: The temperature started at 25 °C and increased by 0.5° every 30 s until the target temperature of 95 °C was reached. FRET was set at 480 and 620 nm, and the fluorescence intensity was monitored.

Isothermal titration calorimetry experiments were performed on an ITC200 (GE Healthcare MicroCal) . All experiments were carried out at 25 °C. About 1 × 0.4 μl followed by 19 × 2 μl of hydroxylamine were injected into a 200 μl enzyme solution. The data were analyzed using the ORIGIN software (MicroCal, Inc).

### HOX activity assays

HOX activities were assayed in 300 μl reaction mixtures in 20 mM Tris–HCl buffer (pH 8.0) containing 10 mM ^15^NH_2_OH, 2 mM NADH, 2 mM NADPH, and 20 μM FAD. About 331 μM DnfA, 3.2 μM DnfB, and 311 μM DnfC were added separately or in combination in the assays, and assays without any of these proteins were run as controls. The reaction was started with the addition of ^15^NH_2_OH (Cambridge Isotope Laboratories, Inc), and the mixture was directly injected into a 10 ml gastight tube, the air of which had been completely replaced by 1:1 of He/O_2_. The reactions were incubated at 30 °C without agitation in the dark for 100 min. The hydroxylamine consumption in the mixtures and ^15^N_2_ release in the headspace were measured. Hydroxylamine was determined by using 8-quinolinol to form the stable 5, 8-quinolinequinone-5-(8-hydroxy-5-quinolylimide) (36). Quantitative detection of ^15^N_2_ was performed by GC/MS (model 7890A/5975C; Agilent) equipped with a CP-Molsieve 5A Plot (25 m × 0.32 mm × 30 μm; Agilent).

To determine the N_2_ production velocities *versus* DnfA concentrations, enzymatic assays were carried out in 300 μl reaction mixtures with initial 1 mM ^15^NH_2_OH, 1 mM NADH, 100 μM FAD, and various concentrations of DnfA (0, 2.5, 5, 7.5, 10, 15, and 20 μM). The reaction mixtures were incubated in 10 ml gastight tubes that were filled with 4:1 of He/O_2_ and at 30 °C for 60 min. To determine the N_2_ production velocity *versus* FAD concentrations, the assays were performed in 1 mM ^15^NH_2_OH, 1 mM NADH, 10 μM DnfA, and 4:1 of He/O_2_. FAD concentrations were used at 0, 1, 10, 50, and 100 μM. Reaction mixtures lacking DnfA were used as controls. Each reaction was incubated at 30 °C for 60 min, and then ^15^N_2_ production was measured. Kinetic assays for DnfA were conducted in 10 μM DnfA, 100 μM FAD, and 1 mM NADH under atmospheric condition, with the concentration of hydroxylamine varied from 15 to 400 μM. Samples from each reaction were taken at 5 min. The *K*_*m*_ and *k*_cat_ values were calculated by nonlinear regression fitting using the Michaelis–Menten equation. Enzymatic assays were performed in triplicate.

### Single-turnover reactions

For single-turnover experiments, 300 μl reaction mixtures in 20 mM Tris–HCl buffer (pH 8.0) were supplemented with 1 mM ^15^NH_2_OH, 1 mM NADH, and various amount of DnfA (15, 30, 60, and 120 nmol). The reactions were conducted in 10 ml gastight tubes that were filled with 4:1 of He/O_2_ and incubated at 30 °C for 60 min. At the end time point, ^15^N_2_ release in the headspace was measured.

### Involvement of O_2_ and H_2_^18^O measurements

O_2_ requirement experiments were carried out using ^18^O_2_ and by detection of the formation of H_2_^18^O. ^18^O_2_/He (10%/90%) was purchased from Newradar Special Gas Co, Ltd. The components of 300 μl reaction mixture and reaction conditions were 331 μM DnfA, 120 μM FAD, 10 mM NADH, 10 mM ^15^NH_2_OH in 20 mM Tris–HCl (pH 8.5), in 10 ml anaerobic tube at interval times. The reaction mixtures were diluted two times, and δ^18^O was detected by isotopic water analyzer (L2130-I; PICARRO, Inc). The concentration of H_2_^18^O was calculated according to the equation: δ (‰) = (Rsample/Rstandard − 1) × 1000. The standard was VSMOW2 (International Atomic Energy Agency). Milli-Q water (Millipore) was used as blank controls.

### EPR measurement

Low-temperature EPR spectra were acquired on a Bruker X-band EMX plus 10/12 spectrometer equipped with an Oxford Instruments ESR 910 continuous helium-flow cryostat. A cylindrical resonator (ER4119hs TE011) was used for EPR data collection. The purified DnfA and the sample reduced by 1 mM dithionite in anaerobic chamber (“reduced” state) were assayed. For samples in mixed-valence state, DnfA was mixed with NADH, FAD, and NH_2_OH, and the reaction was allowed to incubate at 30 °C for 30 min before EPR measurement. About 10% glycerol was added to the protein samples as cryoprotective agent, and each sample was placed into quartz EPR tubes (Wilmad; 707-SQ-250 M, 3 mm inner diameter, 4 mm outer diameter). The EPR tubes were frozen in liquid N_2_ for subsequent EPR measurement. For each sample, multiple scans were accumulated to obtain a good S/N ratio. The experiment parameters are provided in figure legend.

### Immunogold labeling

Fresh HO-1 cells cultivated in HNM medium for 40 h were centrifuged and fixed with 4% formaldehyde and 0.2% glutaraldehyde in 100 mM phosphate buffer (PB) for 2 h. Fixation reagent was removed by washing three times, each for 5 min, in the PB. The cells were embedded in 12% gelatin at 37 °C for 15 min and then placed into ice bath to cure gelatin. The gelatin-embedded cells were cut into small cubes (1–2 mm^3^), transferred into 2.3 M sucrose, and infiltrated overnight at 4 °C on the shaker. Samples were cryosectioned using a cryoultramicrotom. Cryosections (65 nm) were picked up with a drop of 1% methylcellulose and 1.15 M sucrose in PB and transferred to formvar carbon-coated copper 100-mesh grids for immunogold localization. The grid was washed with a drop of PB for 2 min, transferred to PB containing block agent (10% goat serum), and incubated for 30 min. It was incubated with a small drop of mouse-derived antibody against DnfA for 60 min and washed with four drops of PB for 2 min each and then incubated with a small drop of secondary (goat-derived antimouse) antibody-10 nm gold for 60 min and washed with four drops of PB for 2 min each and four drops of water for 1 min each. After the immunogold localization procedure, cryosections were embedded in 1.8% methyl cellulose containing 0.4% aqueous uranyl acetate on ice for 10 min and then air dried. The samples labeled with and without gold nanoparticles were observed using a JEM-1400 transmission electron microscope.

## Data availability

All data relevant to this work are contained within this article and the associated supporting information.

## Supporting information

This article contains supporting information.

## Conflict of interest

The authors declare that they have no conflicts of interest with the contents of this article.
