# Supplementary material for: Identification and characterization of a novel hydroxylamine oxidase, DnfA, that catalyzes the oxidation of hydroxylamine to N2
Source: J Biol Chem. 2022 Aug 13;298(9):102372. doi: 10.1016/j.jbc.2022.102372 (PMC9478400; doi:10.1016/j.jbc.2022.102372)
Supplement: Supplemental Figure S1 [file mmc1.docx]

**Identification and characterization of a novel hydroxylamine oxidase, DnfA, that catalyzes the oxidation of hydroxylamine to N_2_**

Meng-Ru Wu^1, 2†^, Li-Li Miao^1†^, Ying Liu^1†^, Xin-Xin Qian^1^, Ting-Ting Hou^1,2^, Guo-Min Ai^1^, Lu Yu^3^, Lan Ma^1,2^, Xi-Yan Gao^1,2^, Ya-Ling Qin^1,2^, Hai-Zhen Zhu^1^, Lei Du^4^, Sheng-Ying Li^4^, Chang-Lin Tian^3,5^, De-Feng Li^1,2^*, Zhi-Pei Liu^1,2^*, Shuang-Jiang Liu^1,2,4^*

^1^ State Key Laboratory of Microbial Resources and Environmental Microbiology Research Center, Institute of Microbiology, Chinese Academy of Sciences, Beijing, China.

^2^ University of Chinese Academy of Sciences, No.19(A) Yuquan Road, Shijingshan District, Beijing, China 100049.

^3^ High Magnetic Field Laboratory, Chinese Academy of Sciences, 230031, Hefei, China

^4^ State Key Laboratory of Microbial Technology, Shandong University, Qingdao, 266273, China.

^5^ The First Affiliated Hospital of USTC, Division of Life Sciences and Medicine, and Center for BioAnalytical Chemistry, Hefei National Laboratory of Physical Science at Microscale, University of Science and Technology of China, 230026, Hefei, Anhui, China


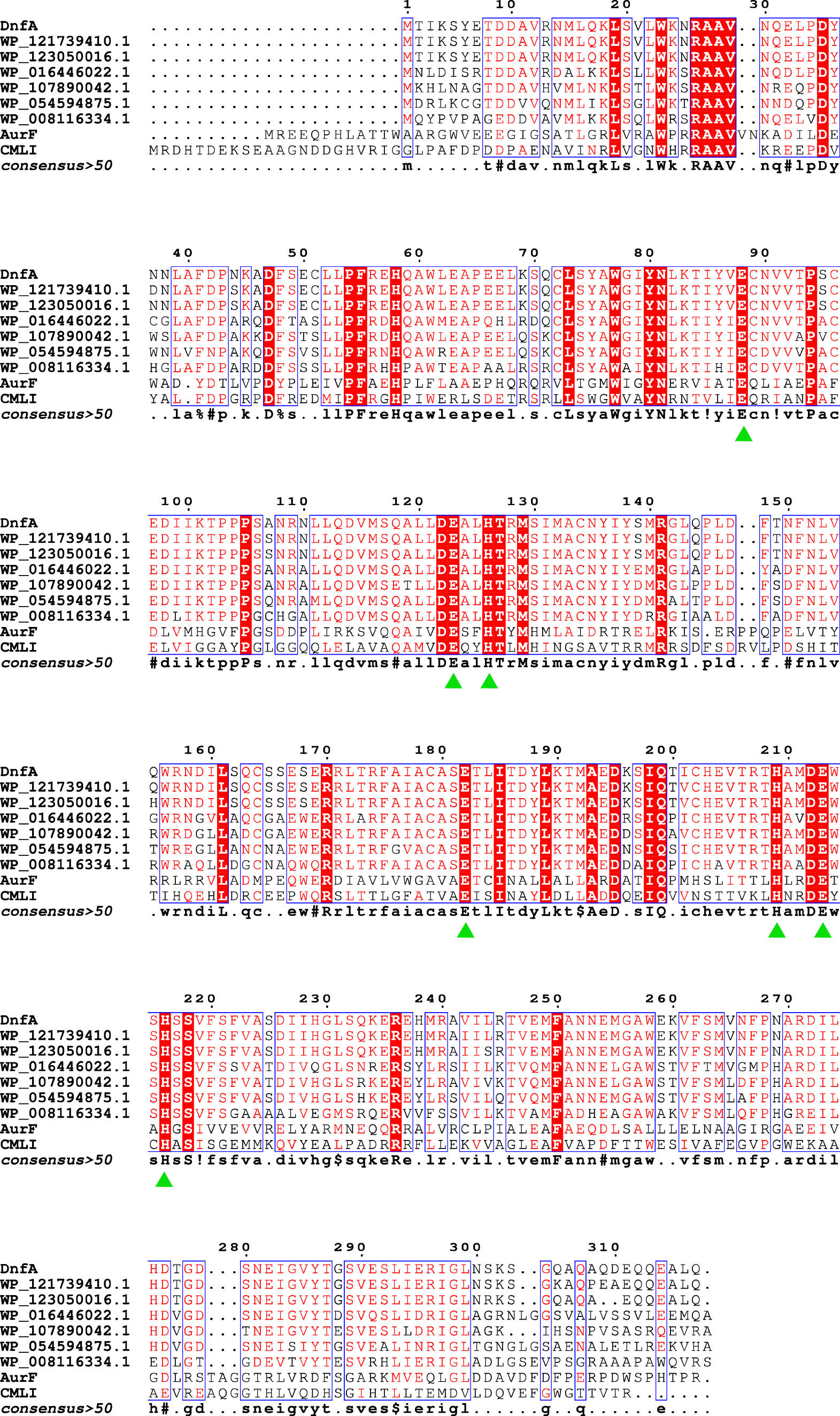


**Figure S1 *In silico* identification of a diiron binding domain within the sequence alignment of DnfA, AurF and other homologous proteins.** Residues involved in diiron binding are indicated by triangles. AurF, *Streptomyces thioluteus* HKI-227; WP_121739410.1, *Alcaligenes aquatilis*; WP_123050016.1, *Alcaligenes faecalis*; WP_016446022.1, *Delftia acidovorans*; WP_107890042.1, *Microvirgula aerodenitrificans*; WP_054594875.1, *Pseudomonas* sp.; WP_008116334.1, *Herbaspirillum* sp. YR522; WP_015032130.1 (CmlI), *Streptomyces venezuelae* ATCC 10712.
